# Prevalence of Hemorrhoids and the Associated Risk Factors Among the General Adult Population in Makkah, Saudi Arabia

**DOI:** 10.7759/cureus.51612

**Published:** 2024-01-03

**Authors:** Raghad O Al-Masoudi, Raghad Shosho, Dhuha Alquhra, Mohammed Alzahrani, Mohanned Hemdi, Lujain Alshareef

**Affiliations:** 1 Faculty of Medicine, Umm Al-Qura University, Makkah, SAU; 2 General Surgery, Prince Sultan Military Medical City, Riyadh, SAU

**Keywords:** online survey, piles, risk factors, makkah, general population, hemorrhoids, prevalence

## Abstract

Introduction: Hemorrhoidal disease is one of the most common benign anorectal conditions. It is described as the symptomatic enlargement and abnormally downward displacement of anal cushions. Its effect on the quality of life of patients is significantly negative and is considered one of the leading causes of lower gastrointestinal bleeding. However, studies that determine the prevalence of and risk factors associated with hemorrhoidal disease are limited. Therefore, this study aimed to evaluate the prevalence of and risk factors for hemorrhoids among the general adult population in the city of Makkah, Saudi Arabia.

Methods: A descriptive cross-sectional study was conducted with a structured, prevalidated questionnaire and was used with some modifications. It was created using Google Forms (Google, Mountain View, CA) and distributed via social media platforms in Arabic along with the English version of each question. All data from the returned survey were encrypted. IBM SPSS Statistics, version 21 (IBM Corp., Armonk, NY) was used to analyze the data.

Results: A total of 400 participants completed the study questionnaire. Regarding the prevalence of hemorrhoids among the general population in Makkah, 64 participants (16%) reported that they were diagnosed with hemorrhoid disorder. The most reported symptoms among participants with hemorrhoids were pain with defecation (76.2%), discomfort (63.5%), and swelling (55.6%).

Conclusion: Hemorrhoids are one of the most common complaints among surgical patients and are more prevalent in men. The risk of hemorrhoids is significantly higher in married women with a history of pregnancy, who are overweight, and who consume low-fiber diets. It is better to practice close follow-up of patients with hemorrhoids to avoid complications, particularly, patients with chronic diseases who are at a high risk.

## Introduction

Hemorrhoidal disease is one of the most common benign anorectal conditions and is described as the symptomatic enlargement and abnormally downward movement of anal cushions, causing venous dilation and prolapse [1-6]. It significantly influences the quality of life of patients and is a leading cause of lower gastrointestinal bleeding. Depending on the location of the dentate line, it is categorized as either internal or external [7]. Additionally, substantial discomfort and disability may result from hemorrhoidal illness [7].

Hemorrhoids are considered a cause of morbidity and can lead to economic and social impacts on the community. They are affected by food, hygiene, and sexual habits, and their symptoms can have physical and psychological impacts [8]. Worldwide, around 50%-85% of people suffer from hemorrhoids. They can affect all genders at any age [9]. The prevalence of hemorrhoids in patients visiting the surgical outpatient department at the University of Gondar Comprehensive Specialized Hospital, Northwest Ethiopia, was 13.1% [8]. The study results were similar to research done in Israel (16%) and Korea (14.4%) [10,11]. The prevalence was higher in Egypt and Austria, which reported a prevalence of 18% and 38.9%, respectively [12,13]. These numbers represent only patients with presumably incidental rather than symptomatic hemorrhoids [8].

The predisposing factors for hemorrhoids are anything that increases intra-abdominal pressure, such as straining during defecation, pregnancy, or obesity. In addition, prolonged sitting and strenuous lifting are considered risk factors for hemorrhoids [14,9].

Studies that determine the prevalence of and risk factors for hemorrhoids in Saudi Arabia are limited. Hence, this study aimed to evaluate the hemorrhoid prevalence and the associated risk factors among the general adult population in the city of Makkah, Saudi Arabia.

## Materials and methods

This cross-sectional study investigated the prevalence of and risk factors associated with hemorrhoids among the general population in Makkah, Saudi Arabia. The participants were selected using a convenience sampling method. The study’s inclusion criteria included any age, gender, or nationality and being a resident of Makkah. Participants who did not agree to the study’s informed consent or did not fulfil the participant criteria were excluded. The data were collected from October to November 2022. Informed consent was obtained from all participants, and all data were kept anonymous. The participants voluntarily completed an online self-administered questionnaire in Arabic and English. The survey was made available through a link shared on social media platforms (Twitter, WhatsApp, and Telegram). The completion of the questionnaire took three to five minutes.

A previously validated questionnaire from previous studies with minor adjustments was reviewed by an expert consultant and was then used in this study [10,11]. The questionnaire was created using Google Forms (Google, Mountain View, CA) and composed of three sections. Section 1 included eight demographic questions about gender, age, nationality, marital status, body mass index (BMI) categories, education level, and family income level. It also contained one question that was intended to ensure that they lived in Makkah. Section 2 consisted of six questions assessing symptoms and past hemorrhoid diagnoses. Finally, Section 3 had seven questions for screening hemorrhoids. Data were analyzed using IBM SPSS Statistics, version 21 (IBM Corp., Armonk, NY). Ethical approval was obtained from the Institutional Review Board of Umm Al-Qura University (HAPO-02-K-012-2022-09-1194).

## Results

**Figure 1:**
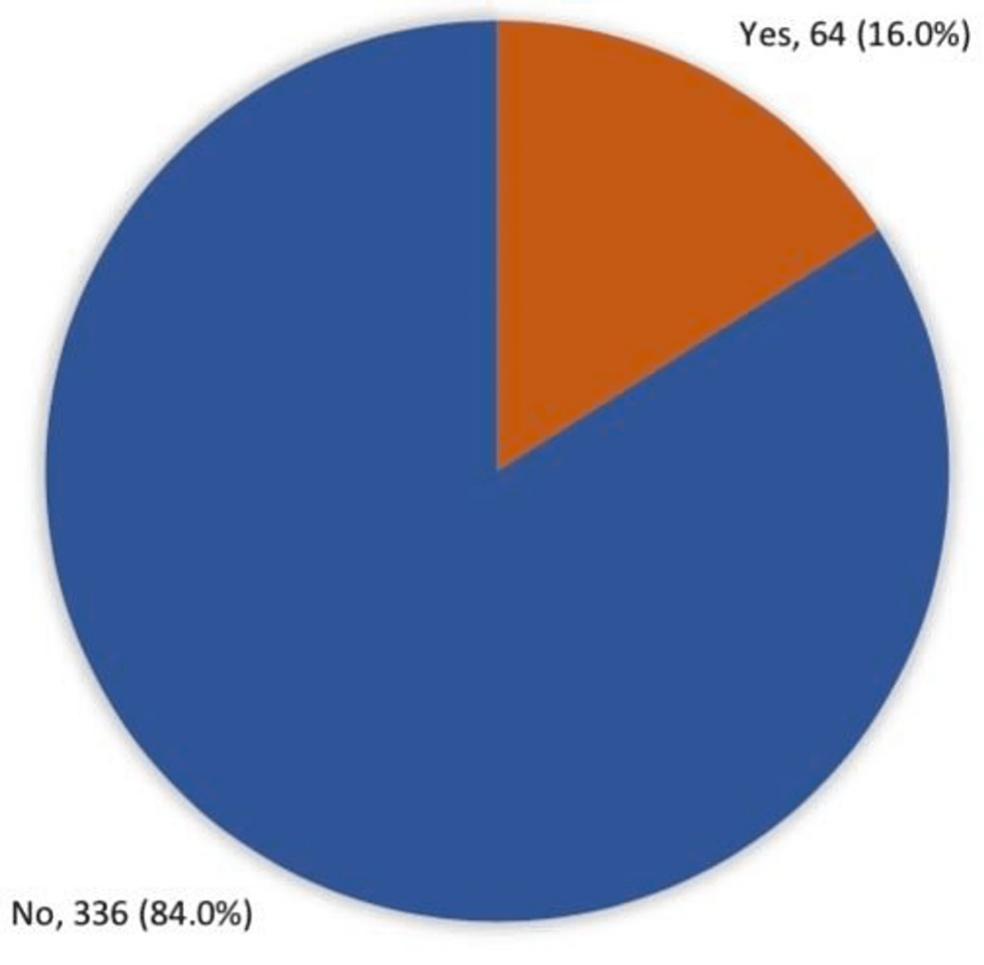
Prevalence of hemorrhoids among the general population in Makkah, Saudi Arabia

In total, 400 participants completed the study questionnaire. Participants’ ages ranged from 18 to more than 50 years, with a mean age of 24.2 ± 13.9 years. A total of 338 (84.5%) were female; 386 (96.5%) were Saudi. A total of 274 (68.5%) were single, and 328 (82%) had a university education or higher. Monthly income exceeding 10,000 Saudi riyal (SR) was reported among 220 (55%) participants. Regarding the prevalence of hemorrhoids among the general population in Makkah, 64 participants (16%) reported being diagnosed with the hemorrhoid disorder (Figure 1).

In this study, the most reported symptoms among participants with hemorrhoids were pain with defecation (76.2%), discomfort (63.5%), and swelling (55.6%). Symptoms mainly occurred during the summer among 15 (24.2%) patients. Twenty-nine (46.8%) reported that they had less than one episode of symptoms per week, while 26 (41.9%) stated that it had happened for one to four weeks. The main symptoms recurred once per year among 24 (38.7%). Regarding hemorrhoid prolapse, 28 (43.8%) cases had prolapse upon straining, which reduced spontaneously. The hemorrhoid clinical data are shown in Table 1.

**Table 1:** Hemorrhoid clinical data of study cases

Clinical data	No.	%
Have you ever suffered from any of these symptoms in the anal region?		
Pain with defecation	48	76.2%
Discomfort	40	63.5%
Swelling	35	55.6%
Oozing	30	47.6%
Itching	30	47.6%
Anal bleeding	23	36.5%
Unusual discharge	9	14.3%
Rectal prolapse	9	14.3%
Have no symptoms	1	1.6%
Season when symptoms occur		
Winter	9	14.5%
Summer	15	24.2%
Autumn	1	1.6%
I do not know	37	59.7%
Duration of episode		
<1 week	29	46.8%
1–4 weeks	26	41.9%
>4 weeks	7	11.3%
Frequency of symptoms		
Once per year	24	38.7%
1–5 times per year	20	32.3%
6–12 times per year	18	29.0%
Hemorrhoid prolapse		
Prolapse upon straining that needs manual reduction	11	17.2%
Prolapse upon straining that reduces spontaneously	28	43.8%
Prolapsed and irreducible hemorrhoids	8	12.5%
No hemorrhoidal prolapse	17	26.6%

Based on demographic data, 52% of participants older than 50 complained of hemorrhoids versus 4.3% of those younger than 20 years, with recorded statistical significance (P = 0.001). In addition, 36% of married persons had hemorrhoids compared to 7.3% of the single group (P = 0.001). Hemorrhoids were detected among 25.5% of persons who were overweight and 19.5% who were obese, compared to 7% of those who were underweight (P = 0.007; Table 2).

**Table 2:** Distribution of study participants’ hemorrhoid frequency by their demographic data SR, Saudi riyal ^*^P < 0.05 (significant). ^$^Exact probability test.

Sociodemographic data	Total		Have you ever been diagnosed with hemorrhoids?				P-value
			Yes		No		
	No.	%	No.	%	No.	%	
Age in years							.001*
<20	47	11.8%	2	4.3%	45	95.7%	
20–30	233	58.3%	18	7.7%	215	92.3%	
30–40	45	11.3%	9	20.0%	36	80.0%	
40–50	50	12.5%	22	44.0%	28	56.0%	
>50	25	6.3%	13	52.0%	12	48.0%	
Gender							.684
Male	62	15.5%	11	17.7%	51	82.3%	
Female	338	84.5%	53	15.7%	285	84.3%	
Nationality							.357^$^
Saudi	386	96.5%	63	16.3%	323	83.7%	
Non-Saudi	14	3.5%	1	7.1%	13	92.9%	
Marital status							.001*
Single	274	68.5%	20	7.3%	254	92.7%	
Married	114	28.5%	41	36.0%	73	64.0%	
Divorced/widow	12	3.0%	3	25.0%	9	75.0%	
Body mass index							.007*
Underweight	43	10.8%	3	7.0%	40	93.0%	
Normal weight	210	52.5%	26	12.4%	184	87.6%	
Overweight	106	26.5%	27	25.5%	79	74.5%	
Obese	41	10.3%	8	19.5%	33	80.5%	
Educational level							.211
High school/below	72	18.0%	8	11.1%	64	88.9%	
University level/above	328	82.0%	56	17.1%	272	82.9%	
Monthly income							.843
<3000 SR	48	12.0%	9	18.8%	39	81.3%	
3000–10,000 SR	132	33.0%	20	15.2%	112	84.8%	
>10,000 SR	220	55.0%	35	15.9%	185	84.1%	

Approximately 21.5% of participants with a family history of hemorrhoidal disease had hemorrhoids, versus 10.8% of others without (P = 0.004; Table 3). Hemorrhoids were also detected in 39.1% of pregnant females versus 7.6% of nulligravida (P = 0.001). Additionally, 24.8% of participants who had a low-fiber diet complained of hemorrhoids, compared to 13.7% of others with a balanced diet and 3.6% of those who received a low-carbohydrate diet (P = 0.002). All cases with Crohn’s disease had hemorrhoids, as did 60% of cases with varicose veins, 42.9% of those with psoriasis, 39.1% with hypertension, and only 9.8% of those with no chronic health problems (P = 0.001).

**Table 3:** Factors associated with hemorrhoids among the general population in Makkah, Saudi Arabia DM, diabetes mellitus ^*^P < 0.05 (significant). ^$^Exact probability test.

Factors	Total		Have you ever been diagnosed with hemorrhoids?				P-value
			Yes		No		
	No.	%	No.	%	No.	%	
Do you have a family history of hemorrhoidal disease?							.004*
Yes	219	54.8%	47	21.5%	172	78.5%	
No	83	20.8%	9	10.8%	74	89.2%	
I don't know	98	24.5%	8	8.2%	90	91.8%	
History of pregnancy, for females							.001*^,$^
Yes	87	25.7%	34	39.1%	53	60.9%	
No	251	74.3%	19	7.6%	232	92.4%	
Smoking							.476^$^
Yes	55	13.8%	7	12.7%	48	87.3%	
No	345	86.3%	57	16.5%	288	83.5%	
Physical activity							.164
Low	176	44.0%	28	15.9%	148	84.1%	
Average	191	47.8%	27	14.1%	164	85.9%	
Athletic	33	8.3%	9	27.3%	24	72.7%	
Types of daily food intake							.002*
Low-fiber diet	141	35.3%	35	24.8%	106	75.2%	
Low-protein diet	56	14.0%	4	7.1%	52	92.9%	
Low-carbohydrate diet	28	7.0%	1	3.6%	27	96.4%	
Balanced diet that contains all nutrition	175	43.8%	24	13.7%	151	86.3%	
Standing during the day							.829
My work requires me to stand	103	25.8%	18	17.5%	85	82.5%	
I move and stand for daily tasks only	219	54.8%	35	16.0%	184	84.0%	
I don’t stand much	78	19.5%	11	14.1%	67	85.9%	
Chronic diseases							.001*^,$^
Hypertension	23	5.8%	9	39.1%	14	60.9%	
Depression	34	8.5%	5	14.7%	29	85.3%	
Chronic constipation	45	11.3%	14	31.1%	31	68.9%	
Hematological disease	4	1.0%	1	25.0%	3	75.0%	
Varicose veins	10	2.5%	6	60.0%	4	40.0%	
DM	22	5.5%	7	31.8%	15	68.2%	
Asthma	25	6.3%	6	24.0%	19	76.0%	
Allergy	51	12.8%	12	23.5%	39	76.5%	
Crohn’s disease	2	.5%	2	100.0%	0	0.0%	
Psoriasis	7	1.8%	3	42.9%	4	57.1%	
None of these	245	61.3%	24	9.8%	221	90.2%	

## Discussion

In this study, we aimed to determine the prevalence of hemorrhoids among the general adult population in the city of Makkah, Saudi Arabia, and the associated risk factors.

According to our study, the prevalence of hemorrhoidal disease was 16% in Makkah, consistent with a study in Northwest Ethiopia that reported a prevalence of 13.1% [8]. Both are less than that reported in Australia, which was 38.9% [12]. This could be attributed to the fact that the data collected in the Australian research were from a colorectal cancer screening area. In a way, hemorrhoids and colorectal cancer have similarities in their symptoms [8].

The current study found that 52% of hemorrhoidal disease patients were older than 50 years. One of the few studies presenting such data found that patients were mostly between 46 and 50 years of age; this is similar to the results of the present study [15]. A 1990 study showed that hemorrhoids most commonly occur between the ages of 45 and 65 [1].

In the course of our investigation, it was observed that the prevalence of hemorrhoids was notably higher among females compared to males. This discrepancy may be attributed to the increased proportion of men actively seeking medical consultation and therapeutic interventions for this condition [1]. Similar to a previous study [15], females made up majority of the hemorrhoidal population. According to another study, there was no gender difference in patients [1]. Additionally, 36% of married persons in our population had hemorrhoids. In particular, 39.1% of married women with a prior pregnancy history had a significantly higher prevalence of hemorrhoidal disease than nulligravida. This is likely due to increased intra-abdominal pressure, pelvic venous congestion, and damage during labor [8].

According to the current study, overweight people had a 25.5% increased risk of hemorrhoids. Similarly, studies elsewhere have supported the association between being overweight and developing hemorrhoids [8]. This likely results from body weight increases and intra-abdominal pressure increases, causing venous congestion of the rectum to rise [10].

Moreover, we found in our study a significant relationship between hemorrhoids and chronic diseases, such as inflammatory bowel disease (IBD), hypertension, and diabetes. These factors play a pivotal role in predisposing individuals to the development, exacerbation, or persistence of hemorrhoids. We suggest chronic inflammation, a hallmark of many chronic diseases, contributes to vascular changes, weakening the integrity of blood vessels. This vascular fragility heightens the susceptibility to vascular anomalies, such as the formation of hemorrhoids. In conditions like IBD, persistent inflammation throughout the gastrointestinal tract can directly impact the rectal area, leading to localized venous engorgement and subsequent hemorrhoidal development. Understanding these connections between chronic diseases and the associated risks or complications, including hemorrhoids, is crucial for holistic patient care to ensure comprehensive management and improved patient outcomes.

This study found that 24.8% of participants who had a low-fiber diet complained of hemorrhoids compared to 13.7% of others with a balanced diet that contained all necessary nutrition. On the other hand, a previous study from Northwest Ethiopia found that 62.53% of people diagnosed with hemorrhoids used to have a low-fiber diet [8].

The most common presentation of hemorrhoids was pain with defection (76.2% cases). This result was almost consistent with a previous study [8]. Discomfort during defections was seen in 63.5% of patients. On the contrary, a recent survey stated that the most common manifestation of hemorrhoids was painless rectal bleeding during defecation with or without prolapsing anal tissue in 71% of cases [9].

This study contributes to our ability to promptly identify those at risk of hemorrhoids and provide an early diagnosis by evaluating the prevalence of hemorrhoids as well as the burden and potential risk factors connected with the condition, in Makkah. However, the study’s cross-sectional methodology has some limitations: this institution-based study’s findings might not reflect the population as a whole, and it is also possible that recall bias was present. Additionally, we cannot confirm whether the patients had only hemorrhoids or other anorectal diseases were present too, which needs a further clinical examination.

## Conclusions

Hemorrhoids are one of the most common complaints among surgical patients and are more prevalent in females than males. The risk of hemorrhoids is found to be higher in old age, married women with a history of pregnancy, those overweight, those with low-fiber diets and patients with chronic diseases such as Crohn’s diseases and varicose veins. Eating a healthy diet rich in fibers and maintaining a healthy weight can aid in relieving symptoms. To avoid complications, it is better to practice hemorrhoid screening in high-risk patients, especially those with chronic diseases. We also recommend increasing the number of community awareness campaigns regarding hemorrhoids and the associated risk factors.

## References

[1] Johanson JF, Sonnenberg A (1990). The prevalence of hemorrhoids and chronic constipation: an epidemiologic study. Gastroenterology.

[2] LeClere FB, Moss AJ, Everhart JE, Roth HP (1992). Prevalence of major digestive disorders and bowel symptoms, 1989. Adv Data.

[3] Janicke DM, Pundt MR (1996). Anorectal disorders. Emerg Med Clin North Am.

[4] Ohning GV, Machicado GA, Jensen DM (2009). Definitive therapy for internal hemorrhoids--new opportunities and options. Rev Gastroenterol Disord.

[5] Riss S, Weiser FA, Schwameis K, Riss T, Mittlböck M, Steiner G, Stift A (2012). The prevalence of hemorrhoids in adults. Int J Colorectal Dis.

[6] Ganz RA (2013). The evaluation and treatment of hemorrhoids: a guide for the gastroenterologist. Clin Gastroenterol Hepatol.

[7] Hong YS, Jung KU, Rampal S (2022). Risk factors for hemorrhoidal disease among healthy young and middle-aged Korean adults. Sci Rep.

[8] Kibret AA, Oumer M, Moges AM (2021). Prevalence and associated factors of hemorrhoids among adult patients visiting the surgical outpatient department in the University of Gondar Comprehensive Specialized Hospital, Northwest Ethiopia. PLoS One.

[9] Ali SA, Shoeb MFR (2017). Study of risk factors and clinical features of hemorrhoids. Int Surg J.

[10] Lee JH, Kim HE, Kang JH, Shin JY, Song YM (2014). Factors associated with hemorrhoids in Korean adults: Korean National Health and Nutrition Examination Survey. Korean J Fam Med.

[11] Al-Humadi AH (2009). Epidemiology of colon & rectal cancer in Iraq. World J Colorectal Surg.

[12] ElBatea H, Enaba M, ElKassas G, El-Kalla F, Elfert AA (2011). Indications and outcome of colonoscopy in the middle of Nile delta of Egypt. Dig Dis Sci.

[13] Ray-Offor E, Amadi S (2019). Hemorrhoidal disease: predilection sites, pattern of presentation, and treatment. Ann Afr Med.

[14] Jensen SL, Harling H, Arseth-Hansen P, Tange G (1989). The natural history of symptomatic haemorrhoids. Int J Colorectal Dis.

[15] Sheikh P, Régnier C, Goron F, Salmat G (2020). The prevalence, characteristics and treatment of hemorrhoidal disease: results of an international web-based survey. J Comp Eff Res.

